# The Impact of Systemic Drug Therapies (Bisphosphonates and Immunosuppressants) on Dental Implant Success: A Clinical Review

**DOI:** 10.31661/gmj.v13iSP1.3666

**Published:** 2024-12-31

**Authors:** Khalid Eisa Aldabeab, Asim Alsuwaiyan

**Affiliations:** ^1^ King Fahd Military Medical Complex, Dhahran Saudi Arabia

**Keywords:** Dental Implants, Bisphosphonates, Immunosuppressants, Osteonecrosis of the Jaw, Osseointegration, Implant Success

## Abstract

Under normal physiological conditions, dental implants are a reliable and effective choice for tooth replacement with high success rates. However, compromised implant success is more closely related to systemic drug therapies like bisphosphonates and immunosuppressants used in patients.This review aims to identify key patterns of implant success and complications among patients undergoing long-term systemic drug therapy and provide clinical recommendations for optimizing outcomes. Bisphosphonates, especially in their intravenous (IV) forms, inhibit osteoclast activity and decrease bone turnover, which may negatively impact osseointegration and increase the incidence of osteonecrosis of the jaw (ONJ). Immunosuppressants are commonly associated with delayed wound healing and a higher risk of infection, complicating implant osseointegration due to their effect on the immune response. Clinicians must adopt personalized approaches to prevent complications in this patient population. Moreover, future studies should aim to explore the long-term effects of systemic drug therapies, particularly regarding dosages, treatment durations, combinations, and newer medications, while investigating drug interactions and dose-response relationships to provide more specific guidelines for clinicians on implant outcomes.

## Introduction

Dental implants have become the most effective solution for tooth replacement due to advances in technology and surgical techniques [1]. However, as more patients require long-term systemic drug therapies for chronic conditions like osteoporosis and autoimmune diseases, there is growing concern about how medications such as bisphosphonates and immunosuppressants impact implant success, particularly through their effects on bone metabolism and immune function [2]. Medications like bisphosphonates, often prescribed for osteoporosis and cancer-related bone conditions, and immunosuppressants, used to manage autoimmune diseases and organ transplants, have been found to disrupt bone metabolism and weaken immune function [3]. Bisphosphonates impair osseointegration by reducing bone turnover, increasing the risk of ONJ in certain patients [4]. Likewise, immunosuppressants impair the body's healing capacity and defense against infections, which can result in delayed osseointegration or an increased risk of implant failure [5]. Given the increasing number of patients on long-term therapies for chronic conditions such as osteoporosis and autoimmune diseases, understanding the role of these drugs on implant outcomes is critical for ensuring safe and effective dental treatments [6].

This review examines the current evidence regarding the impact of bisphosphonates and immunosuppressants on dental implant success, emphasizing their influence on bone healing, immune function, and osseointegration. Also, we seek to offer guidance for clinicians in managing patients on bisphosphonates or immunosuppressants to optimize implant outcomes and minimize complications.

Dental Implant Success Factors

Definition of Success in Implant

Success in dental implantology involves multiple aspects, including mechanical stability, functional performance, aesthetics, and patient satisfaction [7]. While implant survival refers to the implant remaining in place over time, true success also requires the absence of pain, inflammation, bone loss around the implant, and any functional or esthetic issues [8]. A truly successful implant not only integrates well with the surrounding bone but also blends seamlessly with the patient’s natural teeth, supporting normal chewing, speech, and appearance. Clinically, this means stable bone levels around the implant, no signs of inflammation, and the ability to handle regular functional loads [9].

**Figure-1:**
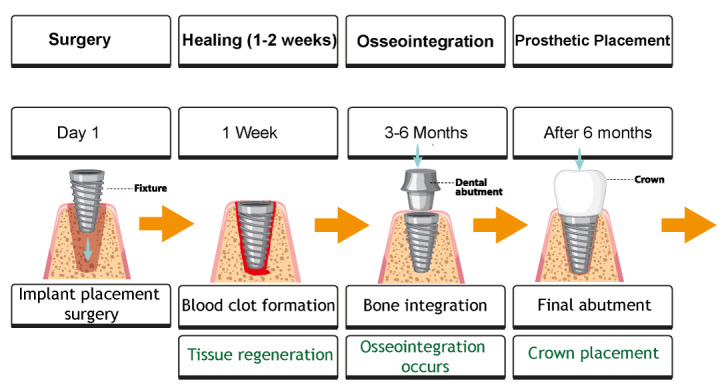
Figure 1. Timeline for the physiological processes involved in dental implant healing and osseointegration. Immediately after implant placement, a blood clot forms at the surgical site (Day 0), initiating the early inflammatory phase (Days 1-7). During this phase, immune cells such as neutrophils and macrophages clear the area of debris and begin the repair process. By the second week (Days 7-14), fibroblasts and osteoblasts begin to proliferate, laying down new connective tissue and bone matrix. The formation of early woven bone occurs around Week 4, providing initial stability. Over the next few months (Weeks 6-12), bone remodeling continues as osteoclasts resorb damaged bone while osteoblasts form mature lamellar bone, gradually enhancing the stability of the implant. Full osseointegration, where the implant is securely anchored to the surrounding bone, typically occurs between 3 to 6 months, depending on patient factors such as bone quality and health.

Figure-1 illustrates the timeline for the key physiological processes involved in dental implant healing, from initial blood clot formation to the maturation of osseointegrated bone.

This progression highlights the critical stages at which osseointegration and bone remodeling occur, factors that clinicians must monitor to ensure long-term implant stability.

## Osseointegration

Osseointegration is fundamental to dental implant stability and success. It involves the direct structural and functional connection between living bone and the implant surface, allowing the implant to become firmly anchored in the jawbone without soft tissue interference [2]. Bone remodeling, a natural physiological process, is key to maintaining this stability. After implant placement, osteoclasts remove old or damaged bone, while osteoblasts generate new bone in response to the mechanical forces exerted by the implant [10]. This continuous process strengthens the bone-implant interface, adjusting it to withstand functional loads and ensuring the implant’s long-term success.

The initial stage of osseointegration involves the formation of a blood clot at the site of implant placement, followed by an inflammatory response that promotes tissue regeneration [11]. Over the following weeks, osteoblasts begin producing new bone matrix around the implant, gradually replacing the provisional woven bone with mature, load-bearing lamellar bone. The surface characteristics of the implant, including roughness, porosity, and biocompatibility, are important in facilitating osseointegration by expanding the surface area and improving osteoblast adhesion. These features help stimulate bone formation, leading to faster and more robust integration with the surrounding bone [12]. Patient factors, including systemic health and bone quality, are also significant determinants of the success of this process [4].

## Healing and Recovery

The healing and recovery process after dental implant placement is a crucial factor in implant success. It occurs in stages, starting with the acute healing phase, where the body reacts to the surgical trauma. In the first few days, inflammatory cells move to the site to kickstart healing, followed by fibroblasts and osteoblasts, which begin to form new connective tissue and bone [13]. This early phase is guided by the body’s inflammatory response, which is vital for clearing debris and encouraging tissue regeneration [10][14].

Over the next few weeks to months, bone remodeling occurs as part of the osseointegration process, and the implant becomes increasingly stable within the bone [15].The typical healing period ranges from 3 to 6 months before loading the implant with a prosthetic restoration. However, in cases where immediate loading protocols are followed or in patients with compromised bone quality, healing may take longer [10][16].

Factors such as smoking, uncontrolled diabetes, and medications like bisphosphonates or immunosuppressants can hinder healing by disrupting normal bone metabolism or raising infection risk [17][18]. Clinicians must thoroughly assess these risks during treatment planning and may need to modify surgical protocols, such as delaying implant placement or extending the healing period to meet individual patient needs [13][17].

## Bisphosphonates

**Table:** Table1. Common Indications for Bisphosphonates and Immunosuppressants

**Drug Class**	**Common Indications**
	- Osteoporosis (postmenopausal, age-related, glucocorticoid-induced)
	- Paget’s Disease of Bone
	- Bone Metastases from cancers (e.g., breast, prostate, multiple myeloma)
**Bisphosphonates**	- Hypercalcemia of Malignancy
	- Osteogenesis Imperfecta (rare genetic disorder)
	- Prevention of Skeletal-Related Events in cancer patients

	- Organ Transplantation (kidney, liver, heart, etc.)
	- Rheumatoid Arthritis
	- Systemic Lupus Erythematosus (SLE)
**Immunosuppressants**	- Psoriasis
	- Crohn’s Disease and Ulcerative Colitis
	- Multiple Sclerosis
	- Pemphigus Vulgaris (autoimmune skin disorder)

Table-1 presents common clinical indications for bisphosphonates, a class of drugs primarily used to treat osteoporosis, bone metastases, and other conditions characterized by excessive bone resorption.

Bisphosphonates inhibit osteoclast activity, reducing bone turnover and maintaining bone density. However, this suppression of bone resorption can hinder bone healing and remodeling, processes critical for the successful osseointegration of dental implants [19].

Bisphosphonates exhibit a unique pharmacokinetic profile characterized by a strong affinity for bone tissue and prolonged retention in the skeleton [20]. A defining feature of bisphosphonates is their long half-life in bone tissue. After a single dose, these drugs can remain bound to bone for years, exerting their anti-resorptive effects over time [21]. For example, oral bisphosphonates like alendronate and risedronate may remain active in the bone for more than 10 years, while intravenous (IV) bisphosphonates such as zoledronic acid allow for annual or biannual dosing, particularly in cancer patients [19].

The prolonged retention of bisphosphonates in bone, while beneficial for preventing fractures, poses challenges in situations requiring bone healing, such as dental implant placement [22].

Even after discontinuing the drug, its effects on bone metabolism persist, which can delay or impair healing. This is especially concerning for high-risk patients, such as those receiving high-dose IV bisphosphonates for cancer treatment, who may require additional precautions during implant procedures.

By limiting bone turnover, bisphosphonates may impair bone remodeling around the implant, which is essential for a stable bone-implant interface [3]. This concern is particularly pronounced in patients on IV bisphosphonates, who are at a higher risk of developing bisphosphonate-related osteonecrosis of the jaw (BRONJ), a severe condition where exposed bone fails to heal, leading to infection, bone destruction, and potential implant failure [2]. Though oral bisphosphonates, commonly used for osteoporosis, present a lower risk of BRONJ, thorough patient evaluation is essential before dental implant surgery.[3] Preventive strategies, such as comprehensive preoperative assessments and minimally invasive techniques, have been shown to reduce the incidence of osteonecrosis [4][6].

Holzinger et al. [23] emphasized the importance of careful treatment timing, finding that patients who received implants during or after bisphosphonate therapy were more likely to develop BRONJ than those treated before therapy began.

The meta-analysis by de Freitas et al., [24] reviewing 15 studies involving 1,339 patients, revealed higher implant failure rates and increased BRONJ incidence in bisphosphonate users compared to healthy patients. This was further corroborated by Sulaiman et al., [25] whose more recent analysis highlighted similarly elevated implant failure rates in bisphosphonate users. These findings underscore the critical role bisphosphonates play in impairing bone healing and integration, stressing the need for tailored implant protocols and patient-specific considerations to optimize outcomes in this high-risk group.

## Immunosuppressants

**Table:** Table2. Clinical Guidelines for Dental Implant Placement in Patients on Systemic Drug Therapies

**Clinical Stage**	**Bisphosphonates**	**Immunosuppressants**
**Pre-Implant Evaluation**	- Assess type, duration, and route of administration (oral vs. IV). - Perform risk assessment for ONJ. - Consider blood tests (e.g., CTX levels) to assess bone turnover. - Collaborate with the patient’s physician if needed.	- Evaluate degree of immune suppression and healing capacity. - Collaborate with the patient’s specialist on timing and dosing of immunosuppressants around surgery. - Assess infection history and wound healing capacity.
**Surgical Considerations**	- Use minimally invasive techniques to reduce trauma. - Avoid or delay implants in high-risk patients (e.g., those on IV bisphosphonates). - Consider prophylactic antibiotics to reduce infection risk. - Extended healing periods before prosthetic loading may be necessary.	- Use atraumatic techniques to minimize tissue damage. - Administer pre- and post-operative antibiotics to reduce infection risk. - Consider adjusting immunosuppressant dosage before surgery (under medical supervision). - Allow for longer healing periods before loading the implant.
**Post-Surgical Care**	- Regular follow-ups to monitor signs of ONJ or implant failure. - Consider extended healing periods before prosthetic loading. - Provide guidance on maintaining oral hygiene to prevent peri-implantitis.	- Monitor for infections or delayed healing. - Longer intervals before implant loading may be needed. - Adjust medication as necessary in collaboration with the patient’s physician to balance immune response and healing.
**Long-Term Monitoring**	- Periodic radiographic evaluations to monitor implant-bone interface and detect early complications. - Regular screenings for signs of ONJ (pain, swelling, exposed bone). - Maintain vigilant oral hygiene and professional cleanings.	- Regular follow-up to check for signs of infection or peri-implant disease. - Long-term antibiotic prophylaxis may be necessary in certain cases. - Ongoing collaboration with medical specialists to adjust medications as needed.

Immunosuppressants, widely used to manage autoimmune diseases (e.g., rheumatoid arthritis, lupus) and to prevent organ transplant rejection, have a significant impact on implant success [26]. Table-1 presents common clinical indications for immunosuppressants, including autoimmune diseases such as rheumatoid arthritis and lupus, as well as post-organ transplantation therapy.

Immunosuppressants, including corticosteroids, cyclosporine, and tacrolimus, function by inhibiting immune pathways, reducing inflammation, and the activity of immune cells like T-cells and macrophages [27]. While this action is necessary for managing autoimmune conditions and preventing organ rejection, it also impairs the body’s ability to heal after dental surgery [28].

They impair wound healing and increase the risk of infection, leading to delayed recovery and potential complications in osseointegration [5]. Clinicians must carefully monitor these patients post-operatively to mitigate risks and ensure successful osseointegration [26].

Immunosuppressants exhibit complex pharmacokinetics, with variability in absorption depending on the specific drug and patient factors [29]. Oral bioavailability is often unpredictable, influenced by factors such as food intake and gastrointestinal conditions [30].

Several studies have underscored the negative impact of immunosuppressants on dental implant success rates. Immunosuppressive medications disrupt the body's natural healing processes and increase susceptibility to infections [26][28]. Chrcanovic et al. [31] reported reduced implant success in organ transplant patients, attributing the decline to chronic immune suppression and delayed tissue healing. An animal study by Sakakura et al. [32] showed that cyclosporin A administration impaired the mechanical retention of dental implants previously integrated into bone, while in a human study, Radzewski et al. [33] suggested that, with proper management, implant placement can be a viable option in immunosuppressed individuals.

## Clinical Considerations

Dental implant procedures require careful planning, risk mitigation strategies, and personalized management for patients on bisphosphonates and immunosuppressants [34]. Table-2 provides a comprehensive clinical guideline for dental implant placement in patients.

The following clinical guidelines provide structured advice and evidence-based recommendations to optimize outcomes and minimize complications in these high-risk patients.

## Pre-implant Evaluation

As general clinical advice, a comprehensive medical history review is essential, with a thorough assessment of the patient’s systemic medication use, including bisphosphonates or immunosuppressants. A detailed understanding of these medications is necessary, as their pharmacological profiles directly influence implant outcomes (Mendes et al., 2019) [35]. Key factors to evaluate include the type of medication, dosage, route of administration, and duration of therapy, as these can directly impact the risk of complications such as ONJ and delayed healing [36][37]. For example, Long-term bisphosphonate therapy, whether administered orally or intravenously, reduces marginal bone resorption and enhances osseointegration. However, IV administration tends to have more pronounced effects, while both routes may negatively impact peri-implant bone remodeling [38]. Moreover, the route of bisphosphonate administration may serve as an independent prognostic factor for advanced-stage MRONJ, irrespective of the dosage or the underlying condition for which the bisphosphonate was prescribed [39].

Additionally, close coordination between the medical team (oral surgeon and the prescribing physicians) is crucial for minimizing the risks related to systemic drug use [40]. This includes consulting with the prescribing physician to understand the patient’s treatment plan and exploring potential adjustments to medication, when feasible, to support better healing and reduce complications. Adjustments or pauses in bisphosphonate treatment before surgery can lower ONJ risks [41].

For instance, in patients on immunosuppressants, coordination with the healthcare team is essential to explore modifications to immunosuppressive therapy. Pre-operative modification of immunosuppressants has been shown to improve outcomes in immunocompromised patients undergoing dental procedures [26].

There are evidence-based recommendations for pre-implant evaluation in patients on systemic drug therapies.

## Evidence-based Recommendations

1. Bisphosphonates:

o Bone Density Assessments (DEXA Scans): According to the American Association of Oral and Maxillofacial Surgeons (AAOMS), assessing bone density is recommended, particularly in patients on long-term bisphosphonates, to evaluate their risk for implant failure [40].

o CTX (C-Terminal Telopeptide) Testing: Studies, including those by Marx et al.,[42] recommend the use of CTX testing to assess bone turnover. CTX levels <100 pg/mL indicate a significantly increased risk for ONJ, necessitating a delay in surgery or reconsideration of implant placement. However, Salgueiro et al, [43] showed that CTX serum levels alone are not a reliable predictor or preventive tool for these complications.

o Risk Assessment for ONJ: IV bisphosphonates pose a high risk of ONJ, particularly in cancer patients [44]. A systematic review by Khan et al. [45] suggests that pre-operative dental evaluations and optimizing bone health before surgery are critical in patients on IV bisphosphonates.

2. Immunosuppressants:

o Adjusting Immunosuppressant Dosage: Evidence from studies such as Chrcanovic et al. [31] suggests that reducing immunosuppressant dosage during the perioperative period reduces the risk of infections and improves healing outcomes. Close collaboration with the patient’s physician is required to adjust the dose of drugs like corticosteroids or calcineurin inhibitors (e.g., cyclosporine).

o Pre-operative Infection Risk Screening: In patients with a history of infections or delayed healing (common in organ transplant recipients), postponing implant placement until infection control is optimized is advised [33].

## Surgical and Post-surgical Considerations

In general, surgical and post-surgical approaches for patients on bisphosphonates or immunosuppressants require special attention due to their increased risk of complications [46].

In these patients, minimally invasive surgical techniques should be prioritized. For bisphosphonate users, the use of atraumatic techniques is essential to minimize bone trauma and avoid disrupting areas with high bone turnover, which could increase the risk of osteonecrosis [47]. Similarly, for immunosuppressed patients, focusing on reducing surgical stress and tissue trauma is crucial, as these patients are more susceptible to delayed healing and infection risks [33].

Extended healing periods are necessary for both bisphosphonate and immunosuppressant patients. Extra time for osseointegration before placing any load on the prosthesis is crucial to ensure proper implant integration with the bone [2][5]. This extended recovery period could take several months longer than usual to reduce the likelihood of complications and support successful implant outcomes [48].

## Evidence-based Recommendations

1. Bisphosphonates:

o Minimize Bone Trauma: Studies have shown that reducing surgical trauma, especially in patients on IV bisphosphonates, can lower the risk of ONJ. According to AAOMS guidelines, surgical procedures should focus on minimally invasive approaches, and flapless surgery may be preferred [40].

o Antibiotic Prophylaxis: Antibiotic prophylaxis offers minimal benefit when used with implant placement and should generally be avoided in most cases [49]. However, In patients undergoing bisphosphonate therapy, Antibiotic prophylaxis before oral surgery is essential for preventing osteonecrosis and supporting healing [50]. Spanou et al., [51] suggested a protocol has proven highly effective in preventing the development of ONJ. They used perioperative IV antibiotic prophylaxis one day prior to surgery and continued throughout the patient's hospital stay. The antibiotic regimen included a single daily IV dose of penicillin (10 million IU). For patients with nonrestorable teeth associated with a purulent infection, an additional dose of metronidazole (500 mg twice a day) was administered. In cases of penicillin allergy, clindamycin (600 mg) was given three times daily as an alternative [51]. However, determining the optimal protocol for the various clinical situations is challenging due to the limited availability of clinical data and randomized controlled trials [50].

o Delay or Avoid Implants: For patients on long-term or high-dose IV bisphosphonates, evidence suggests avoiding implant placement if possible, due to high rates of ONJ and implant failure [24]. If implant surgery is unavoidable, delaying it until after bisphosphonate discontinuation is recommended, when feasible, after consultation with the prescribing physician [25][45].

2. Immunosuppressants:

o Antibiotic Prophylaxis: Given the higher infection risk in immunosuppressed patients, [26] There is sufficient evidence to indicate that administering a single-dose antibiotic (e.g. 2-gram amoxicillin) one hour before surgery may lower the risk of implant failures [52]. Prophylactic antibiotic use before implant surgery offers significant advantages for immunosuppressed patients, with the observed reduction in risk supporting its use in implant dentistry [26]. A recent study found that the use of antibiotics reduced pain in the immediate postoperative period; however, it did not lower infection rates or prevent implant failure in immunocompetent patients [53].

o Dose Adjustment: Adjusting corticosteroid doses around the surgical period can enhance post-surgical healing [26]. Reducing high corticosteroid doses, under medical supervision, improves both soft tissue healing and bone regeneration [54].

## Long-term Monitoring and Management

After surgery, long-term follow-up is critical to ensure the success of the implant, particularly in patients on systemic medications who are at higher risk for complications [55]. Regular follow-up visits every 3 to 6 months during the first year post-implant should be scheduled to monitor implant stability, peri-implant tissue health, and any signs of complications such as bone loss or infection [19].

1. Bisphosphonates:

o Post-Surgical ONJ Monitoring: The AAOMS recommends regular radiographic evaluations for bisphosphonate users, particularly those on IV therapy, to detect early signs of ONJ or compromised osseointegration [40]. Early identification of ONJ improves prognosis and allows for timely intervention [56].

o Prolonged Antibiotic Use in High-Risk Cases: For patients showing early signs of infection or bone exposure, extended antibiotic courses may be required. Long-term antibiotics, combined with minimally invasive procedures, have been shown to reduce ONJ incidence and improve outcomes [57].

2. Immunosuppressants:

o Ongoing Infection Control: For immunosuppressant users, close monitoring of the surgical site for signs of infection or inflammation is essential (Hernández et al., 2019) [55]. Immediate intervention with antibiotics, debridement, or implant revision should be performed at the first signs of complications [53].

o Delayed Loading Protocols: Implant loading should not occur until full healing and osseointegration have occurred. This may extend the loading period by several months, ensuring implant stability and reducing failure risks [33][58].

## Conclusion

This review has demonstrated that both drug classes can significantly influence implant outcomes through their effects on bone metabolism, immune function, and healing processes. Bisphosphonates, especially in their IV form, are associated with an increased risk of ONJ and impaired osseointegration, making it essential for clinicians to carefully assess and manage these risks. Similarly, immunosuppressants contribute to delayed wound healing and increased infection risk, both of which pose challenges for implant stability and long-term success. These factors highlight the necessity for tailored treatment planning and comprehensive risk assessment when placing implants in patients undergoing these systemic therapies. This review highlights the significant impact of bisphosphonates and immunosuppressants on dental implant outcomes. Bisphosphonates, particularly IV formulations, increase the risk of osteonecrosis of the jaw, while immunosuppressants delay healing and raise infection risks, both of which complicate osseointegration. A tailored approach to treatment, including personalized pre-implant evaluations, modified surgical protocols, and ongoing long-term monitoring, is essential to reduce complications and improve implant success rates in these patient populations. Regular assessments, including clinical and radiographic evaluations, help detect early signs of peri-implantitis, bone loss, or other issues, allowing for timely management and better outcomes. Also, the use of prophylactic antibiotics may be considered to minimize infection risk in immunosuppressed patients, while clinicians should avoid unnecessary surgical trauma in patients at high risk for ONJ.

Collaboration between dental professionals and medical specialists is essential to optimize outcomes and minimize complications in these high-risk patients. Close communication with the patient’s physician or specialist can help in adjusting systemic drug dosages or timing around surgery, optimizing immune function, and improving bone healing conditions. This interdisciplinary approach ensures that systemic health and dental procedures are aligned, reducing the risk of complications such as implant failure, infection, or ONJ.

Despite the current understanding of the impact of systemic drug therapies on dental implant success, further research is needed.

Future studies should investigate the long-term impact of these therapies, including dosages, treatment durations, combinations, and newer medications, to establish more specific clinical guidelines.

## Conflict of Interest

The authors declare that they have no known competing financial interests or personal relationships that could have appeared to influence the work reported in this paper.
